# A Case of Interstitial Pneumonia Leading to Respiratory Failure Several Months After COVID-19 Infection

**DOI:** 10.7759/cureus.77153

**Published:** 2025-01-08

**Authors:** Katsuro Kashima, Hiromi Matsuyama, Yusuke Yoshishige, Shouta Nakazono

**Affiliations:** 1 Cardiology, Ibusuki Medical Center, Ibusuki, JPN; 2 Pulmonary Medicine, Kagoshima University, Kagoshima, JPN

**Keywords:** diffuse alveolar damage, interstitial lung disease cause, post-covid-19 complications, pulmonary bulla, pulmonary hypertension

## Abstract

While acute pneumonia is commonly observed in the early stages of coronavirus disease 2019 (COVID-19) infection, isolated cases of interstitial pneumonia have been reported several months later. In this case, interstitial lung disease was identified on a routine follow-up examination five months after infection. Eight months after infection, the patient developed worsening interstitial lung disease, pulmonary hypertension, and worsening symptoms, leading to respiratory failure. Careful follow-up is necessary for patients with COVID-19 who experience progressive pulmonary lesions.

## Introduction

The coronavirus disease 2019 (COVID-19) pandemic has significantly impacted global health, with a substantial proportion of individuals experiencing persistent sequelae, including an increased risk of severe pneumonia. These individuals often exhibit persistent respiratory symptoms and may require ongoing medical management. In most cases of acute pneumonia complicating COVID-19, caused by severe acute respiratory syndrome coronavirus 2 (SARS-CoV-2), ground-glass opacities (GGO) are observed in the lungs from the onset [1,2]. However, some patients develop pneumonia months later, progressing to extensive pulmonary fibrosis and disease worsening [3-5].

In this case, although only mild GGO was noted in both lower lobes during the COVID-19 infection, five months later, follow-up chest computed tomography (CT) revealed bilateral peripheral infiltrates and reticular changes, and echocardiography showed signs of pulmonary hypertension. The patient initially presented with no apparent pulmonary abnormalities post-COVID-19 infection but subsequently developed a rapidly progressive lung disease that led to respiratory failure.

## Case presentation

**Figure 1:**
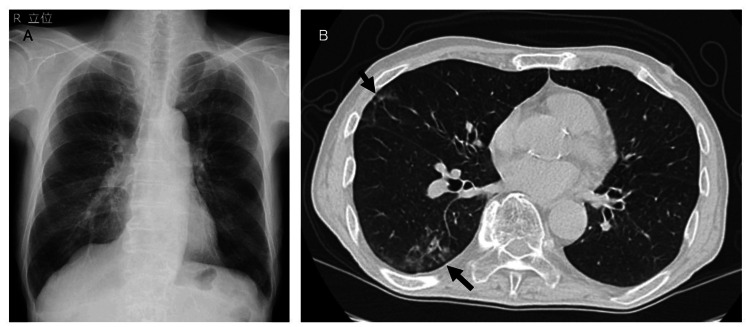
Chest X-rays and CT findings during the acute phase of COVID-19. (A) Chest X-rays showing no abnormalities during the acute phase of COVID-19, (B) while CT revealed mild ground-glass opacities in the right lung (arrows). COVID-19, coronavirus disease 2019; CT, computed tomography

A 77-year-old man with a history of diabetes mellitus and peripheral arterial disease was hospitalized for persistent anorexia for over a week following a COVID-19 infection. He had a 50-pack-per-year smoking history and was unvaccinated against COVID-19. In addition, he was previously employed in a non-industrial setting with no known exposure to asbestos or dust. A previous COVID-19 diagnosis was made via loop-mediated isothermal amplification (LAMP) testing of a nasopharyngeal swab. He did not receive antiviral medication and presented with persistent anorexia despite home care. A diagnosis of post-COVID-19 syndrome was made, the patient received intravenous fluid therapy and nutritional support for anorexia. In the absence of hypoxemia, the patient's appetite improved, and he was discharged home after two weeks. Chest X-rays showed no abnormal findings, but chest CT revealed mild GGO in the right lung field (Figure 1). Laboratory findings demonstrated mild increases in inflammatory marker levels, including a C-reactive protein (CRP) level of 0.61 mg/dL and a white blood cell (WBC) count of 11200/μL, with neutrophils at 9061/μL (Table 1).

**Table 1:** Table 1: Serial changes in blood test results. KL-6, Krebs von den Lungen-6; SP-D, surfactant protein-D; ANA, anti-nuclear antibody; PR3-ANCA, proteinase 3-specific anti-neutrophil cytoplasmic antibody; MPO-ANCA, myeloperoxidase-anti-neutrophil cytoplasmic antibody; QFT, QuantiFERON; IgG, Immunoglobulin G; sIL-2R, soluble interleukin-2 receptor; SS-A, anti-Sjögren’s syndrome A; SS-B, anti-Sjögren’s syndrome B; CCP, anti-cyclic citrullinated peptide; ACE, angiotensin converting enzyme; TNF-α, tumor necrosis factor-α; IL-6, Interleukin-6.

Measure	Reference range	On admission	Five months later	Eight months later
White cells (×1000/μL)	3.3-8.6	11.2	8.3	13.1
Neutrophils (%)	40-74	80.9	67.4	87.3
Lymphocytes (%)	19-48	10.5	11.9	5.9
Monocytes (%)	3.4-9.0	7.9	12.3	5.1
Eosinocytes (%)	0-7.0	0.3	7.9	1.3
Platelets (×1000/μL)	158-348	321	303	335
C-reactive protein (mg/dL)	0.00-0.14	0.61	0.36	1.14
Ferritin (ng/mL)	14.5-332.0	-	27	-
β-D glucan (pg/mL)	11	-	< 5.0	-
KL-6 (U/mL)	<500	-	1708	606
SP-D (ng/mL)	<110	-	-	154
ANA	<40	-	<40	-
PR3-ANCA (IU/mL)	<2.0	-	<0.6	-
MPO-ANCA (IU/mL)	<3.5	-	0.2	-
QFT (IU/mL)	<0.35	-	<0.05	-
IgG (mg/dL)	861-1747	-	670	-
sIL-2R (U/mL)	121-613	-	913	-
SS-A (U/mL)	<10.0	-	<1.0	-
SS-B (U/mL)	<10.0	-	<1.0	-
CCP (U/mL)	<4.5	-	0.5	-
ACE (IU/L)	7.7-29.4	-	19.8	-
TNF-α (pg/mL)	2.27-11.2	-	-	20.1
IL-6 (pg/mL)	<7.0	-	-	24.1

**Figure 2:**
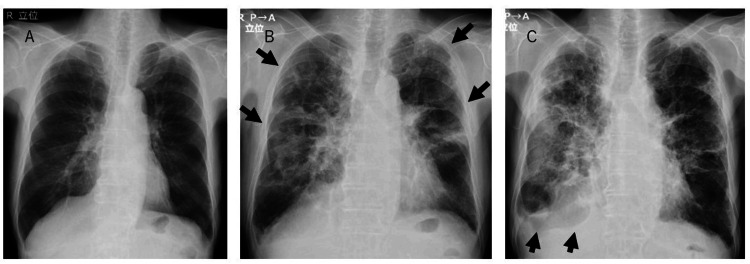
Serial changes on chest radiography. (A) Initial chest X-ray findings in a patient with COVID-19. (B) Five months after COVID-19 infection, bilateral infiltrates developed (arrows). (C) Eight months later, the infiltrates had progressed to involve the entire right lung field with associated pleural effusion (arrows). COVID-19, coronavirus disease 2019

**Figure 3:**
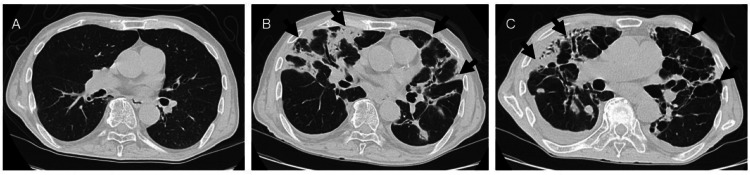
Serial changes on chest CT images. (A) Initially, there were no findings suggestive of pneumonia. (B) Five months later, bilateral diffuse infiltrates developed (arrows), which progressed to bronchiectasis and multiple lung cysts (arrows) eight months after the initial infection (C). CT, computed tomography

Five months later, chest X-rays showed diffuse bilateral infiltrates (Figure 2), while chest CT revealed peripheral infiltrates, reticular changes, and traction bronchiectasis in both lungs (Figure 3). There was no exacerbation of inflammatory marker levels, with CRP at 0.36 mg/dL and WBC at 8300/μL. Krebs von den Lungen-(KL)-6 was elevated at 1708 U/mL, while anti-nuclear antibody (ANA), proteinase 3-specific anti-neutrophil cytoplasmic antibody (PR3-ANCA), myeloperoxidase-anti-neutrophil cytoplasmic antibody (MPO-ANCA), beta-D-glucan, and QuantiFERON (QFT) were negative. The soluble interleukin-2 receptor (sIL-2R) level was slightly elevated, but other markers, such as SS-A antibody, SS-B antibody, anti-cyclic citrullinated peptide (CCP) antibody, and angiotensin-converting enzyme (ACE) were within normal limits (Table 1). Given the absence of new medication use and negative results for collagen vascular diseases, pulmonary fungal infections, pulmonary tuberculosis, and lymphoproliferative diseases, post-COVID-19 organizing pneumonia or interstitial pneumonia was suspected. Echocardiography showed preserved left ventricular function and mild pulmonary hypertension (estimated pulmonary artery pressure: 51 mmHg). In the absence of symptoms or hypoxemia, the patient was managed conservatively without steroid therapy and scheduled for follow-up.

Eight months later, he was rehospitalized owing to persistent anorexia, weight loss of approximately 10 kg, and the onset of dyspnea at rest. Follow-up chest X-rays showed progression of the extensive infiltrates in the right lung field, and pleural effusion (Figure 2). Subsequent chest CT demonstrated marked bilateral diffuse infiltrates, traction bronchiectasis, reticular changes, and cystic changes in the lungs (Figure 3). Despite a decreasing KL-6 level to 606 U/mL, the patient exhibited worsening inflammatory markers (CRP 1.14 mg/dL, WBC 13100/μL, Neu 11436/μL) and elevated inflammatory cytokines such as TNF-α and IL-6 (Table 1). Echocardiography revealed worsening pulmonary hypertension (estimated pulmonary artery pressure 68 mmHg). Given the worsening interstitial pneumonia and the possibility of superimposed bacterial pneumonia, empirical antibiotic therapy with sulbactam/ampicillin (SBT/ABPC) 4.5 g/day was initiated. Oxygen therapy at 1 L/min via nasal cannula was initiated to maintain peripheral oxygen saturation (SpO_2_) at 95%. However, the patient developed acute respiratory failure 10 days after admission and subsequently died.

**Figure 4:**
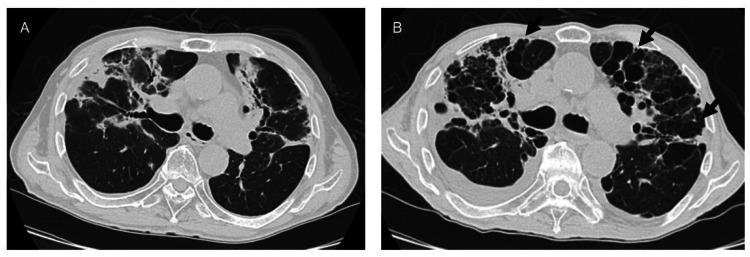
Serial changes in the middle lung field on chest CT images. (A) Image compared with the findings 5 months post-COVID-19 infection; (B) traction bronchiectasis and multiple lung cysts were more prominent in both lung fields eight months later (arrows). CT, computed tomography; COVID-19, coronavirus disease 2019.

A notable finding in this case was the rapid development of bronchial ectasia and multiple pulmonary cysts, which were observed on chest CT five to eight months after COVID-19 infection (Figure 4), suggesting rapid alveolar destruction.

## Discussion

Several cases have been observed in which patients with COVID-19, caused by SARS-CoV-2, have experienced worsening of extensive pulmonary fibrosis and organization several months after infection, leading to severe complications [3-5]. In this case, although only mild GGO was observed in both lower lung fields at the time of COVID-19 infection, extensive lung lesions were confirmed five months later. Chest CT demonstrated multiple, bilateral consolidations, accompanied by traction bronchiectasis and associated parenchymal volume loss. Radiological findings were suggestive of interstitial lung disease. With the exclusion of collagen vascular diseases and drug-induced pulmonary disease, COVID-19-associated interstitial pneumonia was considered. A diagnosis of interstitial lung disease was made based on the presence of traction bronchiectasis and elevated KL-6 levels. Cardiac function was preserved on echocardiography, but mild pulmonary hypertension was noted. Despite reports of steroid efficacy in rapidly progressing post-COVID organizing pneumonia [6], the patient, who was asymptomatic and maintained adequate oxygen saturation, was managed without steroid use. However, eight months post-infection, he was readmitted owing to worsening dyspnea and anorexia. Chest CT at that time revealed progression of restrictive and cystic changes in both lung fields, and echocardiography demonstrated worsening pulmonary hypertension.

There have been reports of the development of emphysema and bullae following COVID-19 infection, sometimes accompanied by pneumothorax [7-11]. In some cases, surgical resection of giant bullae has been reported [12,13]. While some cases suggest that mechanical ventilation contributes to bulla formation, other cases indicate that prolonged coughing without respiratory support may also cause alveolar damage, leading to bulla formation [9]. However, given the absence of mechanical ventilation and refractory cough in this case, mechanical lung injury is considered unlikely.

Pathological findings of COVID-19 associated with acute respiratory distress syndrome include bilateral diffuse alveolar damage with cellular fibromyxoid exudates [14]. Postmortem examination of a COVID-19 patient with acute exacerbation of interstitial pneumonia showed fibrotic lung changes with hyaline membrane formation and inflammatory cell infiltration [3]. While diffuse alveolar damage was the primary cause of death in all 28 affected patients in one study [15], another case demonstrated pulmonary vascular remodeling with intimal and medial thickening in a lung explanted 5 months post-infection [4].

In this case, while KL-6 levels were elevated following COVID-19 infection without accompanying inflammatory findings, the KL-6 level decreased, and inflammatory marker levels increased during the deterioration of respiratory status. It is highly likely that progressive pulmonary fibrosis, accompanied by bronchiectasis, alveolar destruction, and the development of multiple pulmonary cysts, led to respiratory failure due to associated inflammation in the remaining lung tissue. However, it is also possible that the progression of pulmonary hypertension, resulting from pulmonary vascular remodeling, contributed to the respiratory failure.

Although there are reports of pulmonary embolism in post-COVID-19 hypercoagulable states [16], the lack of hypoxemia at the onset of pulmonary hypertension and the concurrent progression of lung field abnormalities and pulmonary hypertension make pulmonary embolism unlikely in this case. The elevated levels of inflammatory cytokines and alveolar epithelial injury markers suggest progressive alveolar destruction following COVID-19 infection [17]. Immune cells have been implicated in the rapid destruction of alveoli in patients who have died due to COVID-19 [18,19], suggesting that future treatments may target the immune response.

Despite the absence of non-invasive positive pressure ventilation, the patient's continued smoking post-discharge may have aggravated pulmonary inflammation, highlighting the importance of intensive smoking cessation counseling. Additionally, the use of corticosteroids, immunosuppressants, and antifibrotic agents should be considered in similar cases. Bronchoscopy was considered at the five-month follow-up visit; however, given the patient's asymptomatic status, the procedure was declined. Retrospectively, it is possible that diagnostic procedures or steroid therapy initiated at this time point may have been beneficial.

## Conclusions

Even in cases where pulmonary lesions were not observed at the initial stage of COVID-19 infection, they appeared several months later. Patients with COVID-19 who develop progressive pulmonary lesions, including interstitial lung disease and pulmonary hypertension, are at risk of respiratory failure and require close monitoring and comprehensive evaluations.
